# Doppler-Defined Pulmonary Hypertension in Sickle Cell Anemia in Kurdistan, Iraq

**DOI:** 10.1371/journal.pone.0162036

**Published:** 2016-09-01

**Authors:** Nasir Al-Allawi, Ameen M. Mohammad, Shakir Jamal

**Affiliations:** 1 Department of Pathology, College of Medicine, University of Duhok, Duhok, Kurdistan, Iraq; 2 Department of Medicine, College of Medicine, University of Duhok, Duhok, Kurdistan, Iraq; 3 Department of Hematology, Azadi Teaching Hospital, Duhok, Kurdistan, Iraq; Vanderbilt University Medical Center, UNITED STATES

## Abstract

To determine the frequency, clinical and laboratory associations of pulmonary hypertension in Iraqi Kurds with sickle cell anemia, a total of ninety four such patients attending a major hemoglobinopathy center in Iraqi Kurdistan were enrolled. All patients were re-evaluated clinically and had their blood counts, HbF, serum ferritin, LDH, renal and liver function assessed. Transthoracic Doppler echocardiography with measurement of tricuspid valve regurgitant jet velocity (TRV) was performed. A TRV in excess of 2.8 m/s was considered for the purposes of this study as indicative of pulmonary hypertension (PH). The prevalence of TRV in excess of 2.8m/s was 10.6%. By univariate analysis: significantly higher reticulocyte count, more frequent blood transfusions and pain episodes were encountered in the PH group as compared to the non-PH group (p = 0.001, 0.045 and 0.02 respectively). Moreover, PH patients had significantly higher mean right atrial area, left atrial size, E wave/A wave ratio and ejection fraction by echocardiography (p = 0.027, 0.037, <0.001 and 0.008 respectively). Except for reticulocyte count none of the other parameters remained significant by multivariate analysis (p = 0.024). In conclusion the current study revealed that pulmonary hypertension is rather frequent among Iraqi Kurds with sickle cell anemia, and identified reticulocyte count as an independently associated parameter with PH in this population. Future prospective studies including right heart catheterization and appropriate medical intervention are warranted.

## Introduction

Sickle cell anemia (SCA) is one of the most important single gene disorders with worldwide distribution. It is due to homozygosity for a single β-globin gene mutation (β6 (A3) Glu→Val, GAG>GTG), and its main clinical hallmarks are vaso-occlusive events and hemolysis, with a consequent variety of complications and end-organ damage [1]. Among its most important pulmonary complications are acute chest syndrome (ACS) and pulmonary hypertension [2]. While most studies have used the non-invasive measurement of tricuspid regurgitant jet velocity (TRV) as an indicator of Pulmonary Hypertension (PH) with respective rates of 20–30% in sickle cell disease [3], using the invasive but "gold standard" method of right heart catheterization documented lower rates of 6.0–10.4% [4–6]. Pathogenesis of PH in sickle cell disease appears to be multifactorial, including: hemolysis induced endothelial dysfunction, chronic hypoxaemia, chronic thromboembolism, asplenia, intravascular sequestration of sickled red cells and iron overload [7]. It is associated with a significant several folds increased mortality rates compared to uncomplicated SCA [8].

The frequency of sickle cell gene varies in different regions of Iraq between 0% and 16%, but clusters in the south and the extreme North of the country [9]. In the latter part of Iraq, most cases are registered in a single center in the Kurdish province of Duhok, where the frequency of the sickle cell gene is 1.2% [10], with around 236 registered SCA patients and a comparable number of Sickle/β-thalassemia syndromes at the only care center dedicated to their care. Despite such significant number of registered sickle cell disease patients in the latter center and the reported high prevalence of PH in SCA in the literature with its associated increased mortality rate, studies addressing its frequency, associated risks and mortality rates in our patients have not yet been initiated. This study is the first study aimed at defining its frequency as well as its clinical and laboratory associations in this part of the world.

## Materials and Methods

This is a cross-sectional study conducted at the inherited blood disorders center in Duhok -Iraq in the period between February 2014 and November 2014. All SCA patients satisfying the inclusion criteria and visiting the latter center during the period of study were enrolled. The diagnosis of SCA (Hb SS) at the latter center is confirmed by a combination of clinical, hematological, family, and if needed molecular studies. All enrolled cases were of Kurdish ethnicity. The inclusion criteria included the following: the patients were confirmed sickle cell anemia (SS) [cases of Sickle/thalassemia syndrome were excluded], were 3 years or older, and that at least 4 weeks had elapsed since acute infection, pain crisis, cerebral vascular accident, acute chest syndrome, blood transfusion or other acute complications of SCA [11]. Patients on hydroxyurea were excluded. Of a total of 236 registered patients with SCA, 86 were on hydroxyurea, 20 were <3 years old, and four were in acute crises at the time of their visit leaving 126 registered eligible patients. Of the latter 94 patients were enrolled (74.6%). The study was approved by the appropriate ethical committee at the Kurdistan board of Medical Specialization (Erbil, Iraq) and an informed written consent was obtained from all participants, or legal guardians (either parent) as appropriate.

### Clinical and Laboratory Assessment

Complete medical history including age, sex, age at diagnosis, age at first transfusion, frequency of transfusion, date of last transfusion, history of splenectomy, history of acute chest syndrome, avascular necrosis of femoral head, leg ulceration, pain crisis necessitating hospital admission, sepsis, priapism, stroke or neurological features were taken from all enrollees. The clinical phenotypes associated with sickle cell anemia were as defined by Ballas (2011) [12]. Physical examination particularly for splenomegaly and for arterial blood pressure was performed for all enrollees.

Investigation performed included: full blood counts (Beckman-coulter hematology analyzer–USA), reticulocyte counts, high performance liquid chromatography (HPLC) for HbF [HLC723-G8 instrument-TOSOH-Japan) and biochemical tests including renal function tests (serum urea and creatinine), liver function tests (Serum total bilirubin, Serum Aspartate and Alanine transaminase [AST and ALT respectively]), serum Lactic dehydrogenase (LDH), and serum ferritin concentrations were measured for all enrolled patients using a biochemistry auto-analyzer (Cobas c501, Roche Diagnostics, HITACHI, Japan).

### Echocardiography evaluations

Transthoracic echocardiography was performed for all enrolled patients using the Vivid 3 (GE) Echo systems (Echolab, USA). The standard two-dimension and M-mode measurements of the cardiac chambers were taken. Color and continuous Doppler study of the cardiac valves were done. Tricuspid Regurgitant Jet Velocity (TRV) was assessed in the apical four chambers, parasternal short axis views, and a minimum of three to five sequential complexes were recorded. For the purposes of this study, pulmonary hypertension was defined as a peak TRV in excess of 2.8 m/s. All echocardiographic measurements were performed according to American society of echocardiography recommendations [13].

### Statistical analysis

All statistical analyses were performed using SPSS software (release 20; SPSS inc, Chicago, IL, USA). Fisher’s exact test was used to compare categorical variables. Continuous variables were represented by means ±SD or medians (range) [depending on whether their distribution is normal or not], and compared using student t-test or Mann Whitney U test, as appropriate. Clinical, echocardiographic and laboratory parameters which had *p* values ≤0.1 by univariate analysis were entered into a logistic regression model for multivariate analysis. *p* values < 0.05 were taken as statistically significant. All *p* values were two tailed.

## Results

### Patients’ Characteristics

The ages of the 94 enrolled patients ranged from 3.0 to 39.0 years, (Median 11.5 years). Forty eight patients were males and forty six were females. The main clinical and laboratory parameters at enrollment are outlined in Tables 1 and 2. It is noteworthy that at the time of their enrollment none of our enrolled patients had a history of leg ulcers, or of documented admission to hospital for sepsis (thus these two parameters were not included in Table 1).

**Table 1:** Clinical and Echocardiographic Parameters in SCA patients overall, and a comparison of those with Pulmonary hypertension (PH) and those without it (Non-PH).

Clinical parameters	Overall (n = 94)	PH(n = 10)	Non-PH(n = 84)	*p* Value Univariate (Multivariate†)
Age (years) [Median (range)]	11.5 (3–39)	11.5 (6–18)	11.5 (3–39)	0.91
Sex:Females (%)	48.9	80.0	45.2	0.078(0.097)
Blood transfusion /year [Median (range)]	5.0 (0–13.0)	7.5 (3.0–13.0)	5 (0–13.0)	0.045(0.577)
Pain crises requiring admission/year[Median (range)]	3.0 (0–12.0)	3.0 (0–12.0)	2.0 (0–11.0)	0.02(0.059)
Priapism (%)	1.1	0	1.2	>0.99
Impalpable spleen/ Splenectomy(%)	62.8	70.0	61.9	0.90
Stroke (%)	3.2	10.0	2.4	0.58
AVN (%)	2.1	0	2.4	>0.99
ACS (%)	1.1	10.0	0	0.21
Systolic BP mm/Hg*	114 ± 8.3	113 ± 6.7	114 ± 8.5	0.708
Left Atrial size (LA)*(mm)	34.1 ± 3.3	36.4±3.7	33.9±3.3	0.027(0.061)
Right Atrial Area (cm^2^)*	16.4 ± 2.0	18.7±1.7	16.1±2.1	<0.001 (0.088)
E wave/A wave ratio*	1.6 ±0.2	1.7±0.2	1.6±0.2	0.037(0.071)
Ejection fraction (EF%)*	63.7 ±4.2	67.2±3.2	63.4±4.3	0.008(0.097)

* Mean ± SD; AVN: Avascular necrosis of femoral head; ACS: Acute Chest Syndrome;

^†^ *p* values for multiple logistic regression.

**Table 2:** Laboratory parameters in enrolled patients and a comparison between PH and non-PH patients.

Parameter	*Mean* ± SD (Except if otherwise specified)			
	Overall (n = 94)	PH (n = 10)	Non PH(n = 84)	*p* Value Univariate (Multivariate†)
Hb (g/dl)	8.5 ± 1.2	8.6 ± 1.0	8.5 ± 1.2	0.76
MCV (FL)	88.7 ± 5.0	88.1 ± 2.3	88.8 ± 5.2	0.69
WBC (x10^9^/L)	14.6 ± 5.2	15.1 ± 3.5	14.5 ± 5.4	0.71
Platelets (x10^9^/L)	399 ± 178	423.3 ± 180.2	396.1 ± 178.8	0.61
Reticulocyte count (%)	17.0 ± 5.7	22.5 ± 4.4	16.4 ± 5.5	0.001(0.024)
HbF (%)*	7.15 (1.1–33.9)	7.1 (1.9–18.9)	7.25 (1.1–33.9)	0.64
ALT (IU/L)*	19 (9–180)	32.5(14–180)	19 (9–110)	0.129
AST (IU/L)*	43 (6–192)	47.5 (24–192)	42.5 (6–117)	0.168
Bilirubin (mg/dl)	4.3 ± 2.9	4.9 ± 2.7	4.2 ± 3.0	0.51
Urea (mg/dl)	17.9 ± 5.6	17.0 ± 4.4	18.1 ± 5.7	0.57
Creatinine (mg/dl)	0.4 ± 0.16	0.35 ± 0.17	0.42 ± 0.15	0.17
Ferritin (ng/ml)*	1060 (35–9170)	1980 (298–6907)	1060(35–9170)	0.144
LDH (IU/L)	628.8 ± 233.1	661.5 ± 295.6	624.9 ± 226.5	0.64

* Median (range);

^†^ *p* values for multiple logistic regression

### Tricuspid Regurigitant Jet Velocity

A total of 10 (10.6%) had a tricuspid regurgitation jet velocity (TRV) in excess of 2.8 m/s, while another 9 (9.6%) had borderline TRV of ≥2.5–2.8 m/s. There was no significant difference between the age of patients with or without PH (*p* value 0.91) [Table 1]; however, it is worth noting that 3/10 patients with PH (30%) were < 10 years old (ages of 6, 6 and 9 years) at the time of enrolment. In relevance to sex, 8 (80%) of 10 patients with PH were females compared to 45.2% females in the non-PH group, however this finding was not significant (*p* = 0.078).

Table 1 shows the main clinical parameters which were assessed in PH and the non-PH groups and shows that none was significantly different, except that patients with PH had significantly higher transfusion rates and higher numbers of acute pain crisis (requiring admission) annually. In relevance to other echocardiographic changes, it was found that patients with PH had significantly higher right atrial area (RA), left atrial size (LA) and E wave/A wave ratio compared to those without PH, and they also had slightly higher mean ejection faction (EF%) [Table 1 and S1 Table].

Table 2 shows the various hematological and biochemical parameters in those with and without PH, and among these parameters only reticulocyte counts was significantly higher (*p* = 0.001) among PH patients. Although serum bilirubin, ferritin and LDH were higher in the PH group, none was found to be statistically significant. There were no differences in renal function tests between the two groups.

Using a multivariate logistic regression model in which all clinical, echocardiographic and laboratory parameters with a *p*≤0.1 by univariate analysis were entered, to compare between those with a TRV ≤ 2.8 and >2.8, only reticulocyte count remained significant (*p* = 0.024).

## Discussion

Our study included 94 SCA patients aged ≥3 years old, all of whom underwent transthoracic echocardiography to determine their TRV as an index of pulmonary hypertension. Most previous studies worldwide have taken a cut-off TRV of ≥ 2.5 m/s as an indicator of pulmonary hypertension [3,14–17]. When the latter cut off point was used, nineteen of our 94 patients (20.2%) would be considered as having PH. This is comparable to a 26% overall rate quoted by a recent meta-analysis with lower rates in children than adults [3]. The rates reported by individual studies, however, varied remarkably from a low 5.2% to a high of 66.0% [14,15, 17–20]. The variations are related to the populations studied, numbers and age ranges of enrolled patients, their disease severity and genotypes. More recently however, a cut-off value of TRV > 2.8m/s was recommended, since it offered higher specificity and positive predictive value for PH when compared to the right heart catheterization, the gold standard for diagnosis [21,22]. Based on the latter recommendation and for the purposes of the current study, a TRV in excess of 2.8 m/s was taken as the cut off point for PH. Ten of the 94 patients (10.6%) satisfied the latter criterion and thus were labeled as PH. This observation is going with previous reports documenting a rate of 10.8–12.9% of PH as deduced by a TRV of >2.8m/s [7,14].

Multiple factors may affect TRV in SCA. Acute but transient elevation in TRV has been observed during uncomplicated pain episodes or acute chest syndrome, which may reflect transient systematic changes [23]. However, this is unlikely in our enrollees as all were in stable state at the time of enrolment and no pain episodes were recorded for at least 4 weeks. Left-sided heart disease with diastolic dysfunction may increase TRV without intrinsic pulmonary vascular disease [19]. This is again not likely in our patients since overt diastolic dysfunction was not seen in any of our patients by echocardiography, though it is prudent to note that the latter modality may underestimate the presence of diastolic dysfunction [24].

The current study showed no significant difference in age in patients with or without PH, which is consistent with several studies focusing on children and adolescents, but is in contrast to those on adults which revealed such a difference [14,25, 26]. This could be explained by the fact that the median age of our enrolled patients is actually 11.5 years. It is important to note that around a third of our PH patients were younger than 10 years, and this stresses the need to screen children (as well as adults) in our region from an early age by echocardiography to detect PH early, when it may be potentially reversible. Earlier studies focusing on children from various populations have made similar recommendations [25, 27]

Females were more frequently affected by PH than males in the current study, however this did not reach the level of significance (p = 0.078). This in contrast to some studies which showed a significant association with male sex in children 10 years aged or older [28], while other studies failed to document an association with gender [14,26,29].

The current study found higher mean reticulocyte count, LDH and bilirubin in those with pulmonary hypertension when compared to those without it, though only the former was statistically significant. These three parameters, especially the reticulocytes count, are important markers of hemolysis. Hemolysis induced endothelial dysfunction has been reported as an important proposed mechanism of PH. Intravascular hemolysis releases free hemoglobin into plasma which in turn scavenges Nitric Oxide (NO) and catalyzes the formation of reactive oxygen and nitrogen species leading to acute and chronic pulmonary vasoconstriction [7]. It also releases erythrocyte Arginase, which acts on L-Arginine changing it to Ornithine, leading to depletion of plasma Arginine, the obligate substrate for NO synthase. Thus intravascular hemolysis leads to decrease production and increase destruction of NO [7,20]. Other workers also demonstrated the association of PH with hemolytic markers in SCD [2,29], and similar to the current study several authors found that the reticulocytes, but not other hemolytic markers like LDH or degree of anemia were associated with PH [7,27,28]. It is important to note that among various clinical, echocardiographic and laboratory variables found to be significant by univariate analysis only the reticulocytes (%) remained so by multivariate analysis, making it an important independent predictor of PH.

Two clinical parameters were found to be associated with PH by univariate analysis, namely the frequency of transfusions and the number of pain crises requiring admission annually. The former maybe related to the fact that patients with PH tend to be symptomatic and thus are more likely to receive transfusions. A similar observation was made by Hagar *et al* (2008) in adults and by Lee *et al* (2009) in children [25,26]. On the other hand, the higher number of pain episodes in the current study is similar to that seen by Colombatti *et al* (2010) [27], but is in contrast to other studies which disputed the association [14,26]. Both these clinical parameters were insignificant by multivariate analysis.

The occurrence of pulmonary hypertension among patients with sickle cell seems to be independent of overt left ventricular systolic or diastolic dysfunction as there was no evidence of compromise of either cardiac function in our study. Although it should be noted that the slightly higher LA in the PH group may reflect some relative, though not overt, diastolic dysfunction compared to non-PH group. This is in consistence with previous studies [14,29]. On other hand, the slightly higher EF values, though still within normal ranges, in our patients with highest TRV is an interesting observation requiring further scrutiny, and is in contrast to some studies, though it is in agreement with others, probably related to the difference of ages of cases in different studies [14,29].

A limitation of the current study is that it did not include right heart catheterization, though this limitation is shared by many previously published studies. Moreover, the positive predictive value for PH could have been further improved by performing the six minute walk (6MW) test or serum N-terminal pro-Brain Natriuretic Peptide (NT-pro-BNP), both non-invasive, as proposed by previous researchers [4,11]. The latter point should be taken in consideration in future studies.

## Conclusion

This study documented for the first time the prevalence of PH among SCA patients in Northern Iraq. Pulmonary hypertension as defined by TRV in excess of 2.8 m/s was associated with an important indicator of hemolysis, namely reticulocyte count, which may serve as a predictor of PH in SCA. The study also demonstrated the need to screen children < 10 years for PH, since one third of our PH cases were in this age category, and early diagnosis may limit complication. Future prospective studies that may incorporate NT-pro-BNP and 6MW tests as well as echocardiography are recommended.

## Supporting Information

S1 Table The Echocardiographic findings in 94 Sickle cell anemia Iraqi Kurdish patients. EA: E wave/A wave ratio; EF: Ejection Fraction; LA: left atrial size; RA: right atrial area; TRV: Tricuspid Regurigitant Jet Velocity.
(XLSX)
